# Altered Activity of Lateral Orbitofrontal Cortex Neurons in Mice following Chronic Intermittent Ethanol Exposure

**DOI:** 10.1523/ENEURO.0503-20.2021

**Published:** 2021-03-02

**Authors:** D. A. Gioia, J. J. Woodward

**Affiliations:** Department of Neuroscience, Medical University of South Carolina, Charleston, SC 29425

**Keywords:** alcohol use disorder, fiber photometry, GCaMP6, OFC, operant self-administration

## Abstract

The lateral orbitofrontal cortex (LOFC) is thought to encode information associated with consumption of rewarding substances and is essential for flexible decision-making. Indeed, firing patterns of LOFC neurons are modulated following changes in reward value associated with an action outcome relationship. Damage to the LOFC impairs behavioral flexibility in humans and is associated with suboptimal performance in reward devaluation protocols in rodents. As chronic intermittent ethanol (CIE) exposure also impairs OFC-dependent behaviors, we hypothesized that CIE exposure would alter LOFC neuronal activity during alcohol drinking, especially under conditions when the reward value of ethanol was modulated by aversive or appetitive tastants. To test this hypothesis, we monitored LOFC activity using GCaMP6f fiber photometry in mice receiving acute injections of ethanol and in those trained in operant ethanol self-administration. In naive mice, an acute injection of ethanol caused a dose-dependent decrease in the frequency but not amplitude of GCaMP6f transients. In operant studies, mice were trained on a fixed ratio one schedule of reinforcement and were then separated into CIE or Air groups. Following four cycles of CIE exposure, GCaMP6f activity was recorded during self-administration of alcohol, alcohol+quinine (aversive), or alcohol+sucrose (appetitive) solutions. LOFC neurons showed discrete patterns of activity surrounding lever presses and surrounding drinking bouts. Responding for and consumption of ethanol was greatly enhanced by CIE exposure, was aversion resistant, and was associated with signs of LOFC hyperexcitability. CIE-exposed mice also showed altered patterns of LOFC activity that varied with the ethanol solution consumed.

## Significance Statement

These studies demonstrate that, in intact mice, lateral orbitofrontal cortex (LOFC) neurons are acutely inhibited by alcohol and become hyperexcitable following chronic intermittent ethanol (CIE) exposure. Furthermore, we report that unique patterns of LOFC neuronal activity occur during alcohol seeking and consumption. Interestingly, these patterns of activity are modulated following CIE exposure, particularly when the rewarding properties of the alcohol solution are modulated through adulterations with quinine (aversive) or sucrose (appetitive). Conversely, control animals have considerably more stable patterns of LOFC activity following exposure to air. These unique effects of CIE exposure on LOFC activity likely contribute to the development of excessive alcohol consumption and behavioral inflexibility that are associated with the onset of alcohol dependence.

## Introduction

Alcoholism is a chronically relapsing disorder that is characterized by excessive alcohol consumption and a lack of behavioral flexibility surrounding alcohol consumption. Indeed, addicted individuals will continue to consume excessive amounts of alcohol despite the development of a variety of negative social, legal, and health related consequences. Furthermore, this behavioral inflexibility presents a significant barrier to the implementation of efficacious treatment strategies. Accordingly, it is important to obtain a deeper understanding of the brain systems that underly this behavior and to learn how chronic exposure to alcohol impairs neurons in these regions.

Behavioral flexibility associated with alcohol drinking can be examined experimentally in rodent models using reward devaluation protocols where stimulus-reward relationships are unexpectedly manipulated (Hamilton and Brigman, 2015). Previous studies have shown that mice will self-administer high levels of alcohol and then reduce consumption when the bitter tastant quinine is added to the alcohol solution or when mice are pretreated with lithium chloride to produce illness which is then associated with alcohol drinking (Lopez et al., 2014; den Hartog et al., 2016). However, animals exposed to repeated cycles of chronic intermittent ethanol (CIE) vapor show impaired behavioral flexibility during these procedures and continue to drink high levels of alcohol even following reinforcer devaluation (Lopez et al., 2014; den Hartog et al., 2016).

The lateral orbitofrontal cortex (LOFC), a subregion of the prefrontal cortex, plays important roles in associative reinforcement learning and modifying behavioral responses when action outcome relationships are changed or devalued (Teitelbaum, 1964; Baltz et al., 2018). Accordingly, lesions or inactivation of the LOFC, impair the acquisition of associative learning, and the modification of learned behaviors following reward devaluation (Schoenbaum et al., 2002; West et al., 2011; Gourley et al., 2013; Gardner et al., 2017; Panayi and Killcross, 2018). Furthermore, studies using *in vivo* electrophysiology have shown that LOFC neurons fire in response to reward predictive cues, signaling the predictive value of the reward and also following reward consumption providing information about the actual value of the reward (Schoenbaum et al., 1998, 2003; Padoa-Schioppa and Assad, 2006). Additionally, LOFC neurons integrate reward value over time and modify their firing in accordance with new contingencies (Riceberg and Shapiro, 2017).

Using slice preparations, we have previously shown that the intrinsic excitability of LOFC neurons is reduced by low concentrations of ethanol (Badanich et al., 2013; Nimitvilai et al., 2020) and that withdrawal from CIE results in enhanced current-evoked spiking (Nimitvilai et al., 2016, 2018). Furthermore, lesions to or chemogenetic inhibition of the LOFC promotes escalation of alcohol drinking in CIE-exposed mice (den Hartog et al., 2016). Considering the important role of this brain region in behavioral flexibility and its sensitivity to the acute and chronic effects of alcohol, it is not surprising that CIE also impairs performance on LOFC-dependent reversal learning (Badanich et al., 2011) and reward devaluation tasks (den Hartog et al., 2016).

Despite these findings, little is known regarding how chronic alcohol exposure impairs the way that neurons in the LOFC modulate their activity when there is a change in the stimulus-outcome relationship during alcohol self-administration. The studies in this manuscript address this issue and use fiber photometry and a mouse model of alcohol dependence to assess LOFC neuronal activity during operant self-administration of ethanol in the absence and presence of aversive or rewarding tastants.

## Materials and Methods

### Animals

Male C57BL/6J mice at four weeks of age were obtained from Jackson labs and allowed one week to acclimate to the Medical University of South Carolina (MUSC) housing facility before surgery and operant ethanol self-administration. Lights in the housing facility were set on a reverse light dark schedule with lights off at 9 A.M. and on at 9 P.M. All drinking and fiber photometry studies were conducted during the dark cycle. All procedures were approved by the MUSC Institutional Animal Care and Use Committee and are consistent with NIH guidelines concerning the use of animals in research. We chose to focus these initial studies on male mice as they show robust and reproducible increases in alcohol drinking and self-administration following exposure to repeated cycles of CIE. In contrast, there is significant variability in CIE-induced drinking in female mice with many studies including our own (Zamudio et al., 2021) showing little to no change in drinking following CIE exposure. This may reflect the higher baseline levels of drinking usually observed in female mice or other as yet identified factors.

### Surgery

Mice were deeply anesthetized with isoflurane vapor (Penlon vaporizer; 1 l/min, 3% induction, 1.5–2% maintenance) and 300 nl of AAV1-CaMKII-GCaMP6f (Addgene) was injected into the LOFC (AP: +2.4; ML: −1.35; DV: −2.4 mm). A custom-made optical fiber and ferrule (400-μm diameter patch cord in a 1.25-mm ferrule; Thorlabs) were implanted at these coordinates and secured in place using Herculite. Mice recovered in their home cage for 7 d before beginning lever-press training. All mice were inspected postmortem to ensure proper viral expression and implant location. Any mice with inaccurate placements or lacking viral expression were removed from the study. Mice with damaged fiber optic implants but accurate expression were excluded from photometry analysis because of poor signal quality but were included in drinking microstructure analysis (*n* = 3 Air, *n* = 2 CIE).

### Postprandial drinking

Access to food and water was regulated throughout this experiment to increase ethanol consumption using a postprandial drinking protocol similar to that described previously (King et al., 2017). Mice were food restricted until 1 h before the start of the daily drinking session. They were then allowed to eat *ad libitum* for 1 h before and 1 h after the drinking session. Water was continuously available until 1 h before drinking sessions at the same time that mice were given access to food. The weight of the mice did not significantly change as a result of this feeding paradigm.

### Operant ethanol self-administration

Mice self-administered ethanol on a fixed ratio one schedule in daily 30-min sessions, Monday through Friday. During these sessions, mice were placed in sound-attenuated Med Associates boxes, with a fan and house light turned on at the beginning of each session. Following an active lever press, mice received auditory (tone) and visual (house light off) cues for 1.5 s while 20 μl of 10% ethanol (v/v) solution were delivered to a drinking well using calibrated Med Associates pumps. The active lever had a 1.5-s time out during the presentation of the cue and reward delivery. A second inactive lever was included in the box that when pressed produced no cues or delivery of the ethanol solution. Drinking at the alcohol well was monitored using Med Associates Lickomoters and licking microstructure was analyzed by grouping bursts of licks into drinking bouts. A drinking bout was determined to be at least three licks occurring with <1 s in between, similar to Renteria et al. (2020).

### Fiber photometry

**Figure 1.**
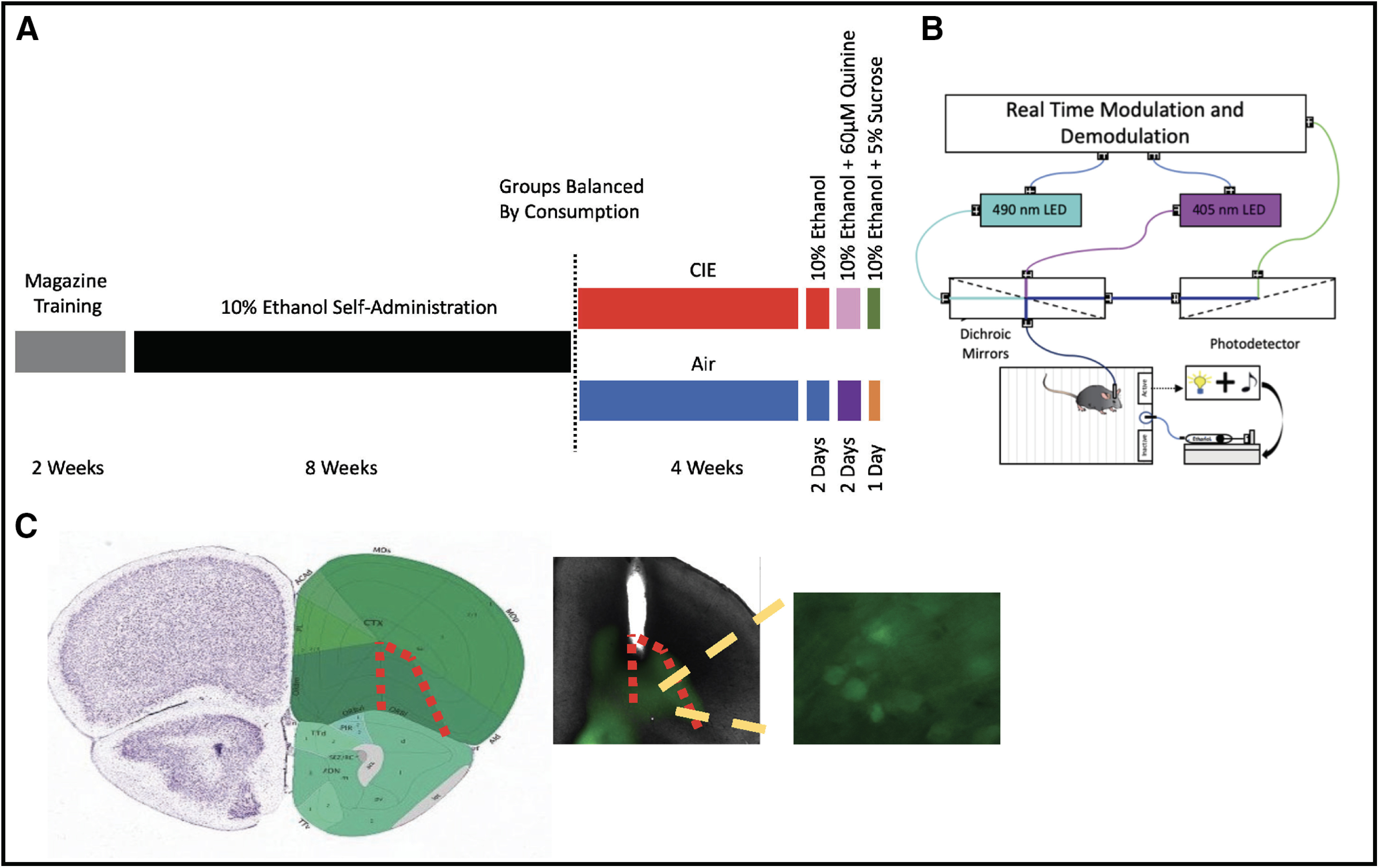
Experimental design. ***A***, Timeline of operant training, drinking sessions, and CIE/Air exposure. ***B***, Schematic of fiber photometry set up with operant drinking chamber. ***C***, Image from Allen Brain Atlas showing orbitofrontal cortex and location of fiber photometry recordings. Inset, Representative image of fiber optic track and AAV-mediated GCaMP6f expression.

Data were acquired using custom-built imaging equipment based on that described by the Deisseroth laboratory (Lerner et al., 2015), with modifications. Illumination was provided by 405- and 490-nm fiber collimated LEDs (Thorlabs; 30 μW per channel) connected to a four-port fluorescence mini-cube (Doric Lenses). The combined LED output passed through a 400-μm optical fiber (0.48 NA) pigtailed to a rotary optical swivel (Doric Lenses) and connected to the implanted fiber using a ceramic sleeve or pinch connector (Thorlabs). Emission light was focused onto a photodetector (Newport, model 2151; DC low setting) and sampled at 6.1 kHz by a RZ5P lock-in digital processor (Tucker-Davis Technology) controlled by Synapse software. Excitation light was sinusoidally modulated at 531 Hz (405 nm) and 211 Hz (490 nm) via software control of an LED light driver (Thorlabs). Real-time demodulated emission signals from the two channels were acquired at a frequency of 0.93084 kHz and stored off-line for analysis (Fig. 1*B*). Lever-presses, head entries and licking at the drinking port were time-locked to fiber photometry data using inputs from the Med Associates hardware to the digital processor. Data were processed using custom-written functions in MATLAB (MathWorks) software. The signals for each channel were first fitted to a polynomial versus time curve and then subtracted from one another to calculate the Δ*F*/*F* time series. Video of the test sessions were recorded using a C930e webcam (Logitech) affixed to the top of the operant chamber.

### Experimental timeline

Figure 1*A* shows the timing of experimental manipulations, operant self-administration training and fiber photometry sessions. Mice (five weeks old) received surgery and were given one week to recover before beginning ethanol drinking. Mice were then exposed to 30-min non-contingent ethanol drinking sessions for two weeks to establish stable drinking of 10% ethanol before the establishment of operant responding for 10% ethanol. Mice then self-administered 10% ethanol for eight weeks; fiber optic recordings were taken weekly for each mouse. Mice were then separated into Air or CIE groups. These groups were counterbalanced by ethanol consumption during baseline drinking sessions. The CIE protocol was similar to those used in previous studies (Becker and Lopez, 2004; Zamudio et al., 2021). Briefly, all mice were injected with the alcohol dehydrogenase inhibitor pyrazole (1 mmol/kg) before being exposed to either air or ethanol vapors for 16 h/d, Monday through Friday. Mice underwent forced abstinence over the weekend. This pattern of exposure continued for four consecutive weeks. Beginning 72 h after the final ethanol or air-exposure mice, underwent five daily self-administration sessions during which they consumed different solutions. On the first 2 d mice drank 10% ethanol to re-establish drinking behavior, on the next 2 d, they drank 10% ethanol + 60 μm quinine, and on the last day they drank 10% ethanol + 5% sucrose (Fig. 1*A*). During drinking sessions, licking at the ethanol port was monitored using lickometer circuitry and this data were used to evaluate the drinking microstructure including lick rate and bout size.

### Statistical analysis

Data were analyzed using Prism 8 software (GraphPad Inc.). Average values were obtained from all drinking bouts or lever presses in each session and used for comparison across group and drink type. Outliers were identified using Grubb’s test. When appropriate, repeated measures (RM) one-way ANOVAs with the Geisser–Greenhouse correction were used to compare effects of different drinking solutions on calcium signals within groups. In other instances, RM two-way ANOVAs with the Geisser–Greenhouse correction were used to compare data between groups with the repeated measure being within subject values. *Post hoc* tests corrected for multiple comparisons were conducted following all ANOVA analyses with significant main effects or interactions. In all cases, values were considered statistically significant when *p* < 0.05.

## Results

### LOFC activity during intraperitoneal injection of alcohol

**Figure 2.**
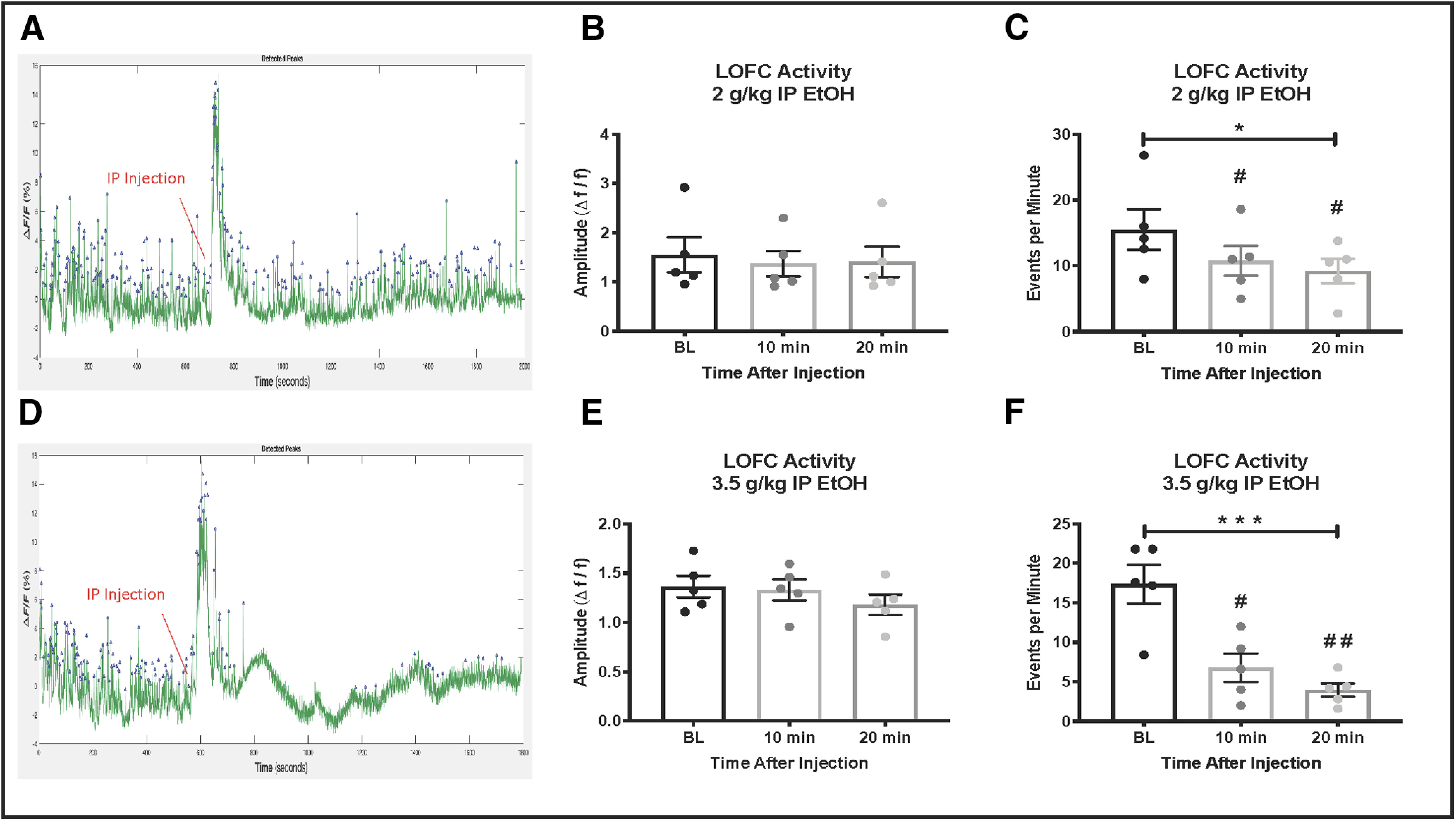
LOFC activity during intraperitoneal ethanol injection. ***A***, Example trace of 30-min LOFC GCaMP6f activity before and after intraperitoneal injection of 2 g/kg ethanol. The ethanol injection causes a large spike in activity followed by recovery. ***B***, 2 g/kg ethanol does not alter the amplitude of GCaMP6f events. ***C***, 2 g/kg ethanol significantly decreases the frequency of GCaMP6f-mediated events. ***D***, Example trace of 30-min GCaMP6f activity before and after intraperitoneal injection of 3.5 g/kg ethanol. The ethanol injection causes a large spike in activity followed by a sustained reduction in activity. ***E***, 3.5 g/kg ethanol does not alter the amplitude of GCaMP6f events. ***F***, 3.5 g/kg ethanol injection inhibits the frequency of GCaMP6f events; * indicates significant main effects, *p < 0.05, ***p < 0.001, # indicates significant *post hoc* tests, #*p* < 0.05, ##*p* < 0.01.

Results from our previous studies using *ex vivo* slice preparations showed that low concentrations of ethanol reduce current-evoked spiking of LOFC neurons (Badanich et al., 2013). In the present study, we used *in vivo* fiber photometry to examine whether ethanol also reduces LOFC activity in the awake, freely moving animal. Activity of the calcium indicator GCaMP6f was recorded in the homecage over a 10 min baseline period and then mice were given an intra-peritoneal injection of ethanol followed by an additional 20 min of recording. Mice showed spontaneous calcium transients during the baseline period that were detected and quantified using the *findpeaks* function in MATLAB. There was a sharp increase in the amplitude of the GCaMP 6F signal at the time of ethanol injection followed by a decrease in activity within 10 min (Fig. 2*A*, 2*D*), consistent with the time course of distribution of ethanol to the brain following intraperitoneal injection (Nurmi et al., 1994). A dose of ethanol (2 g/kg, 20% v/v) that is associated with locomotor stimulation (Smoothy and Berry, 1985) reduced the frequency of GCaMP6f transients by ∼25% (RM one-way ANOVA, *F*_(1.156,4.624)_ = 13.73, *p* = 0.015, Dunnett’s multiple comparisons test was significant at 10 min: q = 4.91, *p* = 0.014 and 20 min: q = 3.69, *p* = 0.036, *n* = 5; Fig. 2*C*). A higher dose of ethanol (3.5 g/kg, 20% v/v) that produces sedation (Aguayo et al., 2014) reduced the frequency of GCaMP6f peaks by ∼80% (RM one-way ANOVA, *F*_(1.991,7.965)_ = 19.74, *p* < 0.001, Dunnett’s multiple comparisons test was significant at 10 min: q = 4.68, *p* = 0.016, and 20 min: q = 5.82, *p* = 0.008, *n* = 5; Fig. 2*F*). Ethanol had no effect on the amplitude of GCaMP6f events at either the 2 g/kg (RM one-way ANOVA, *F*_(1.181,4.725)_ = 2.52, *p* = 0.18, *n* = 5; Fig. 2*B*) or 3.5 g/kg dose (RM one-way ANOVA, *F*_(1.095,4.380)_ = 2.50, *p* = 0.18, *n* = 5; Fig. 2*E*). These data are consistent with those from our slice electrophysiology studies showing an acute inhibitory effect of ethanol on LOFC neuron firing (Badanich et al., 2013).

### Operant ethanol self-administration

Mice were trained to lever press for 10% ethanol under an FR1 schedule of reinforcement. After eight weeks of baseline drinking in daily 30-min sessions, they were separated into CIE or Air groups counterbalanced by baseline ethanol consumption. Mice were then exposed to four consecutive weeks of CIE or air exposure, as described in previous studies (Becker and Lopez, 2004; den Hartog et al., 2016; Nimitvilai et al., 2016; Zamudio et al., 2021). CIE-exposed animals reached an average blood ethanol concentration of 217.08 mg/dl (SEM = 8.75). Three days after the final CIE exposure, mice returned to operant cages and underwent a week of self-administration trials. During the first 2 d, they lever pressed for 10% ethanol to re-establish self-administration behaviors. On days 3 and 4, the ethanol solution was adulterated with the bitter tastant quinine (60 μm) while on day 5, mice self-administered 10% ethanol sweetened with 5% sucrose.

**Figure 3.**
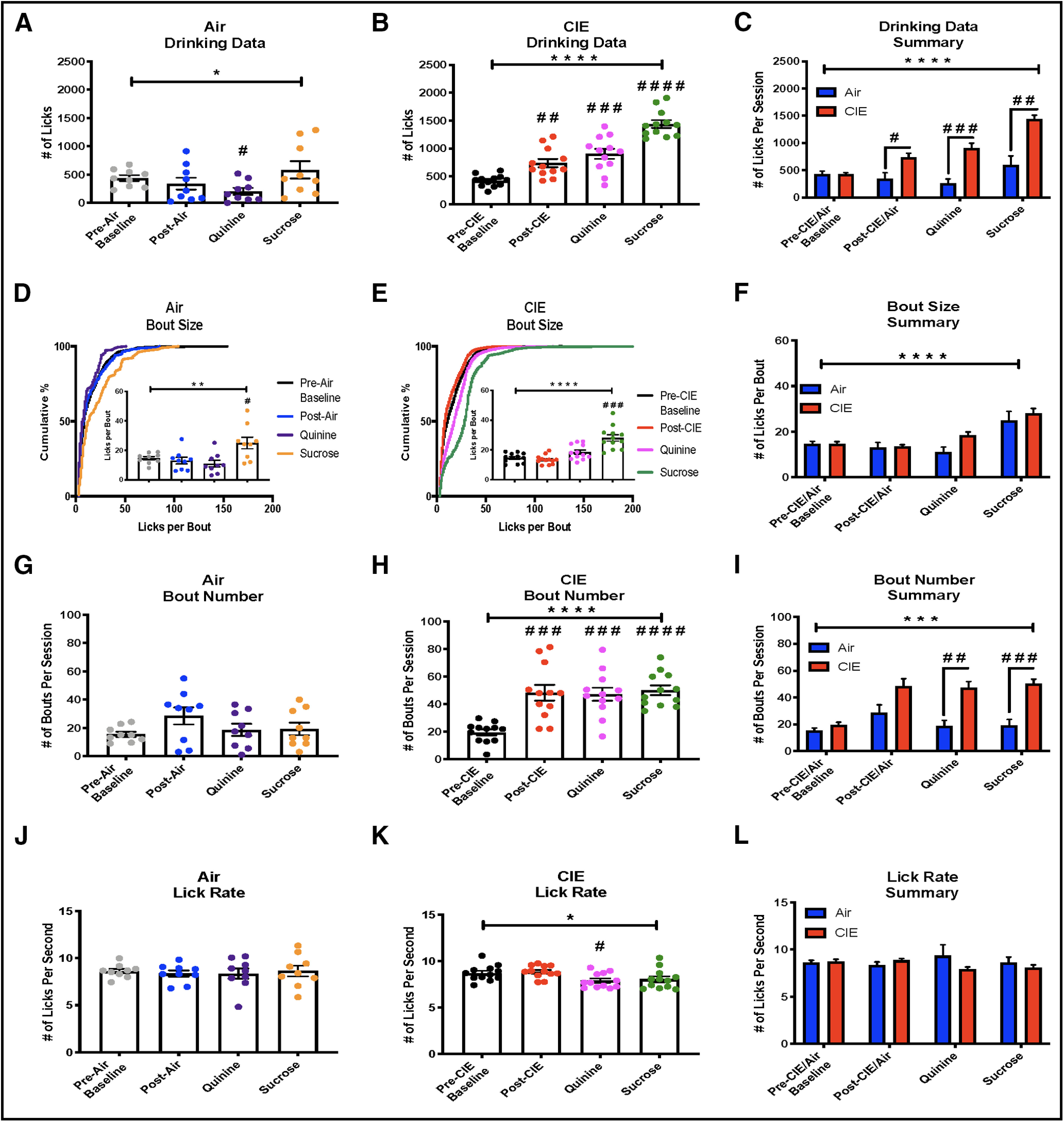
Drinking microstructure. ***A***, Air-exposed mice are sensitive to the devaluing effects of 60 μm quinine and consumed significantly less of that solution compared with baseline. ***B***, Following CIE exposure, mice consumed significantly more ethanol, ethanol+quinine, and ethanol+sucrose solutions. ***C***, Compared with air-exposed mice, CIE mice consumed significantly greater amounts of the ethanol, ethanol+quinine, and ethanol+sucrose solutions. ***D***, In air-exposed mice, drinking bout size changed depending on the drinking solution, posttests revealed that this was driven by larger bouts during ethanol+sucrose drinking. ***E***, In CIE-exposed mice, drinking bout size changed depending on the drinking solution, posttests revealed that this was driven by larger bouts during ethanol+sucrose drinking. ***F***, Bout sizes were significantly larger in CIE animals, posttests revealed that this was driven by increased bout sizes in ethanol+quinine and ethanol+sucrose drinking. ***G***, The number of bouts during a drinking session was not significantly different in any of the drinking solutions for air-exposed mice. ***H***, CIE mice initiated significantly more drinking bouts following CIE exposure when drinking any of the ethanol solutions. ***I***, Compared with air-exposed mice, CIE mice initiated significantly more drinking bouts following CIE exposure when drinking ethanol+quinine or ethanol+sucrose. ***J***, Lick rates during drinking sessions were not significantly different in any of the drinking solutions for air-exposed mice. ***K***, Lick rates were significantly different in CIE-exposed mice with slower rates during ethanol+quinine. ***L***, When compared with air-exposed mice, lick rates were not significantly different during any of the drinking solutions; * indicates significant main effects or interactions *p < 0.05, **p < 0.01, ***p < 0.001, ****p < 0.0001, # used for significant *post hoc* tests #*p* < 0.05, ##*p* < 0.01, ###*p* < 0.001, ####*p* < 0.0001.

Consistent with previous literature (Lopez and Becker, 2005), mice exposed to repeated cycles of CIE significantly increased their consumption of 10% ethanol (RM one-way ANOVA, *F*_(1.951,21.47)_ = 38.7, *p* < 0.0001, Dunnett’s multiple comparisons test significant for post-CIE drinking: q = 4.38, *p* = 0.0029 *n* = 12; Fig. 3*B*). Additionally, CIE-exposed animals consumed significantly more 10% ethanol adulterated with 60 μm (Dunnett’s q = 6.51, *p* = 0.0001; Fig. 3*B*) and sweetened with 5% sucrose (Dunnett’s q = 12.93, *p* < 0.0001; Fig. 3*B*) relative to baseline drinking. Conversely, air-exposed mice showed no change in ethanol consumption following air exposure, but consumed significantly less quinine+ethanol and no difference in sucrose+ethanol (RM one-way ANOVA, *F*_(1.551,12.41)_ = 4.57, *p* = 0.04 *n* = 9, Dunnett’s multiple comparisons test was significant for quinine: q = 3.7, *p* = 0.015; Fig. 3*A*). Figure 3*C* summarizes these findings and shows that CIE-exposed mice had higher levels of consumption of each solution as compared with Air control mice (RM two-way ANOVA, significant main effects of CIE: *F*_(1,19)_ = 30.82, *p* < 0.0001, and drink: *F*_(2.499,47.47)_ = 24.46, *p* < 0.0001, significant interaction: *F*_(3,57)_ = 12.05, *p* < 0.0001. Sidak’s multiple comparisons test was significant for post-CIE/Air: *t* = 2.98, df = 14.85, *p* = 0.037; quinine: *t* = 5.29, df = 18.93, *p* = 0.0002; and sucrose: *t* = 4.76, df = 10.9, *p* = 0.0024).

The microstructure of alcohol drinking in Air-exposed and CIE-exposed mice was assessed by analyzing the number of licks per bout, the number of bouts per session and the number of licks per second. Bout size in air-exposed mice varied significantly across the different drinking solutions (RM one-way ANOVA, *F*_(1.666,13.33)_ = 12.35, *p* = 0.0014, Dunnett’s multiple comparisons test was significant for sucrose+ethanol: q = 3.12, *p* = 0.035; Fig. 3*D*). Likewise, bout sizes were significantly changed following CIE exposure, primarily because of increased drinking of the appetitive solution (RM one-way ANOVA, *F*_(1.648,18.13)_ = 24.4, *p* < 0.0001, Dunnett’s multiple comparisons test was significant during sucrose+ethanol drinking, q = 5.86, *p* = 0.0003, and trended toward significance with quinine+ethanol drinking (Dunnett’s q = 2.54, *p* = 0.068; Fig. 3*E*). When comparing between groups, bout sizes were not significantly altered by CIE exposure (RM two-way ANOVA, significant main effect of drink: *F*_(1.755,33.35)_ = 32.41, *p* < 0.0001; but not CIE: *F*_(1,19)_ = 1.768, *p* = 0.20; or interaction: *F*_(3,57)_ = 2.45, *p* = 0.073, Sidak’s multiple comparisons test was insignificant across all solutions, *p* > 0.05; Fig. 3*F*).

There were no significant differences in the number of bouts per session in air-exposed mice for the different drinking solutions (RM one-way ANOVA, *F*_(2.102,16.82)_ = 3.34, *p* = 0.058; Fig. 3*G*). However, bout number was significantly increased following CIE exposure (RM one-way ANOVA, *F*_(1.628,17.9)_ = 23.34, *p* < 0.0001, Dunnett’s multiple comparisons test was significant at post-CIE: q = 5.34, *p* = 0.0007, quinine: q = 6.58, *p* = 0.0001, and sucrose: q = 11.04, *p* < 0.0001; Fig. 3*H*). The number of bouts per session was greater in CIE-exposed mice compared with air-exposed mice (RM two-way ANOVA, significant main effects of CIE: *F*_(1,19)_ = 17.7, *p* = 0.0005, and drinking solution: *F*_(2.075,39.43)_ = 17.71, *p* < 0.0001, significant interaction: *F*_(3,57)_ = 7.75, *p* = 0.0002, Sidaks multiple comparisons test was significant for quinine: *t* = 4.46, df = 18.94, *p* = 0.0011, and sucrose: *t* = 5.45, df = 16.68, *p* = 0.0002; Fig. 3*I*).

Finally, there were no differences in lick rate across drinking solutions for air-exposed animals (RM one-way ANOVA, *F*_(2.203,17.62)_ = 0.34, *p* = 0.74). CIE treatment produced a small but significant effect on lick rate (RM one-way ANOVA *F*_(2.207,24.27)_ = 4.89, *p* = 0.014; Fig. 3*J*) that was driven by a decrease in lick rate during the ethanol+quinine drinking sessions (Dunnett’s multiple comparisons test, q = 3.48, *p* = 0.013). However, this effect did not reach statistical significance when lick rates between Air-treated and CIE-treated mice were compared with one another (RM two-way ANOVA non-significant main effects of CIE: *F*_(1,19)_ = 0.63, *p* = 0.4369, and drink: *F*_(1.851,35.17)_ = 0.28, *p* = 0.74, interaction *F*_(3,57)_ = 2.41, *p* = 0.076; Fig. 3*L*). Collectively, these findings demonstrate that CIE exposure alters both the consumption and drinking structure of ethanol containing solutions.

### LOFC activity during ethanol drinking

**Figure 4.**
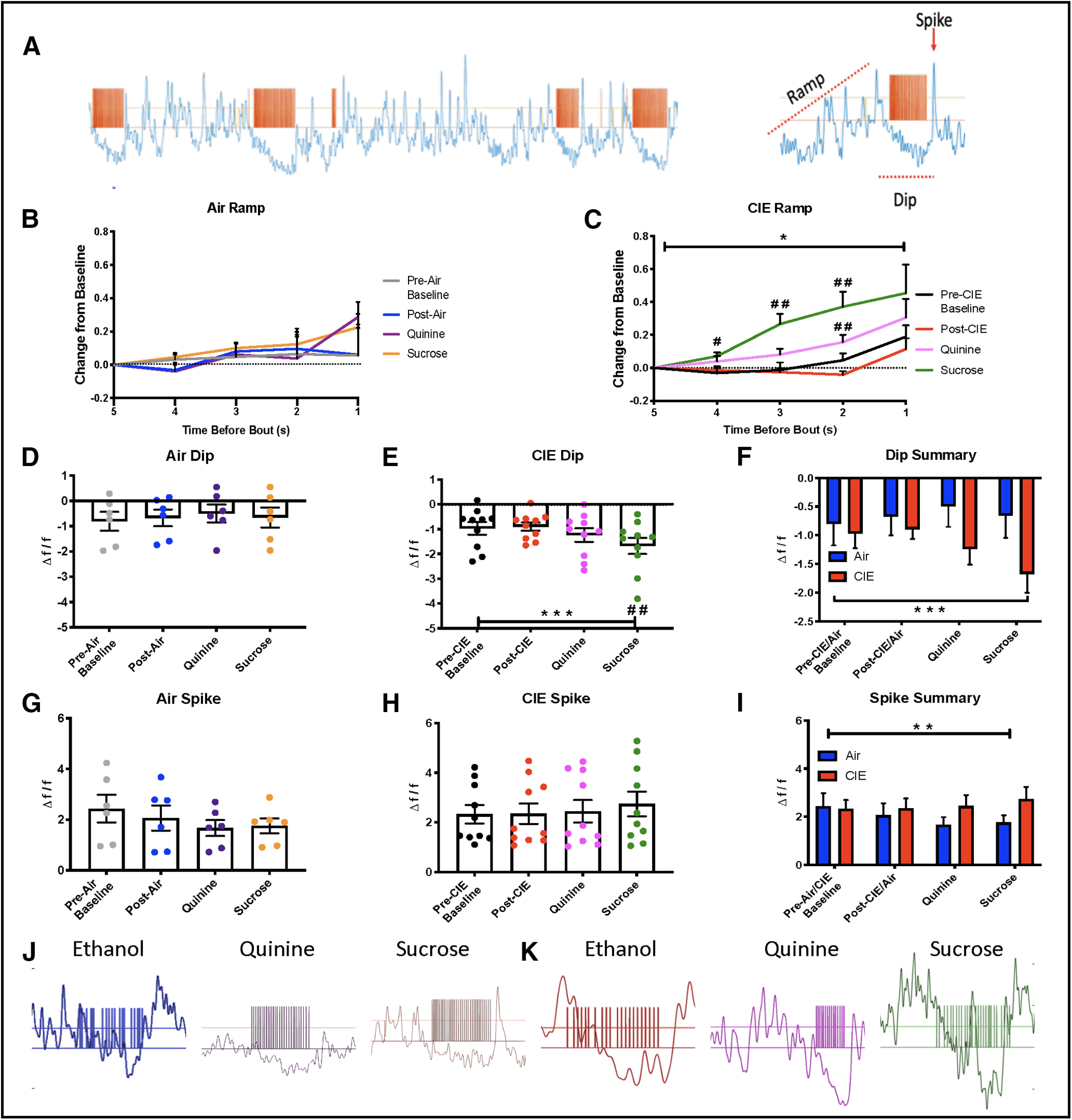
LOFC activity during operant ethanol self-administration. ***A***, Representative trace showing LOFC GCaMP6f signal during operant drinking. Orange ticks are TTL pulses generated during licking at the ethanol port. A characteristic pattern of activity surrounded drinking bouts, with an increase in activity before drinking (Ramp) a decrease in activity during drinking (Dip) and an increase in activity after a drinking bout (Spike). ***B***, No significant differences in the ramp up of activity preceding a drinking bout in air-exposed animals. ***C***, The prebout ramp is significantly different in CIE animals with an earlier and larger increase in activity preceding drinking bouts with ethanol+quinine and ethanol+sucrose. ***D***, There are no significant differences in the size of the dip during drinking bouts in air-exposed animals. ***E***, The size of the dip is significantly different in CIE animals with a significantly larger dip during ethanol+sucrose drinking. ***F***, There was a significant main effect of drink and a significant interaction showing larger dip size in CIE animals. ***G***, The size of the after drinking bout spike was not significantly different across drinking sessions in air-exposed mice. ***H***, The size of the after-drinking bout spike was not significantly different in CIE-exposed mice. ***I***, There was a significant interaction indicating increased spike size in CIE animals. ***J***, Exemplar traces of LOFC GCaMP6f activity in from the same air-exposed mouse during sessions of ethanol (left), ethanol+quinine (middle), and ethanol+sucrose (right) solutions. Exemplar traces of LOFC GCaMP6f activity in the same CIE-exposed mouse during sessions of ethanol (left), ethanol+quinine (middle), and ethanol+sucrose (right) solutions; * indicates significant main effects or interactions *p < 0.05, **p < 0.01, ***p < 0.001, # used for significant *post hoc* tests #*p* < 0.05, ##*p* < 0.01.

The genetically encoded calcium indicator GCaMP6f was combined with fiber photometry to measure LOFC neural activity during the same self-administration sessions described above. Analysis of these recordings under baseline conditions revealed three main characteristics of LOFC activity associated with drinking bouts. As illustrated in Figure 4*A*, LOFC activity increased immediately preceding the initiation of a drinking bout (Ramp), decreased during active drinking (Dip), and rebounded immediately after the bout ended (Spike). These characteristic patterns of activity are generally similar to those reported by other investigators during sucrose drinking in rats (Moorman and Aston-Jones, 2014) and non-human primates (Tremblay and Schultz, 1999) and ethanol drinking in non-dependent rats (Hernandez and Moorman, 2020).

Differences in the magnitude of these three features from the preexposure baseline drinking sessions were calculated for each of the post-CIE/Air drinking sessions. CIE-exposed mice had a significant increase in the size of the ramp-up in GCaMP6f signal, which occurred earlier in CIE-exposed mice during drinking of either of the novel ethanol solutions (RM two-way ANOVA, significant main effects of time: *F*_(1.142,10.28)_ = 7.06, *p* = 0.021, and drink: *F*_(1.626,14.63)_ = 8.64, *p* = 0.004, and a significant interaction, *F*_(2.893,26.04)_ = 3.28, *p* = 0.038, Dunnett’s multiple comparisons test revealed significant differences during sucrose drinking at 2 s: q = 4.26, *p* = 0.0055, 3 s: q = 4.01, *p* = 0.0079, and 4 s: q = 2.90, *p* = 0.044 before the bout and during quinine drinking at 2 s: q = 4.16, *p* = 0.0063 before the bout; Fig. 4*C*). Conversely, air-exposed mice had no significant differences in the prebout ramp in any of the drinking sessions (RM two-way ANOVA, non-significant main effects of time: *F*_(1.925,9.625)_ = 1.41, *p* = 0.29, and drink: *F*_(1.772,8.861)_ = 0.49, *p* = 0.61, and no interaction: *F*_(2.256,11.28)_ = 1.32, *p* = 0.31; Fig. 4*B*).

CIE-exposed mice also showed a significantly larger dip in activity during the drinking bout that was most pronounced during ethanol+sucrose drinking (RM one-way ANOVA, *F*_(1.929,17.36)_ = 11.02, *p* = 0.0009, Dunnett’s multiple comparisons test was significant during sucrose drinking, q = 4.05, *p* = 0.0075, and was trending toward significance during quinine drinking, q = 2.59, *p* = 0.07; Fig. 4*E*). Air-exposed mice had no significant differences in dip size during any drinking sessions (RM one-way ANOVA, *F*_(1.339,6.695)_ = 1.44, *p* = 0.29; Fig. 4*D*). When these effects were compared across both groups, there was a significant interaction between CIE and drinking solution (two-way ANOVA, main effects of drink: *F*_(2.4,33.6)_ = 4.29, *p* = 0.017, CIE: *F*_(1,14)_ = 1.65, *p* = 0.22, interaction: *F*_(3,42)_ = 6.65, *p* = 0.0009, Sidak’s multiple comparisons test revealed significant no significant differences across drinking sessions; Fig. 4*F*). Together, these results indicate that exposure to CIE enhances the size of the dip in LOFC activity when tastants are added to ethanol solutions.

In both Air-exposed and CIE-exposed mice, there were no within group differences in the size of the postbout spike in GCaMP6f signal [air: RM one-way ANOVA, *F*_(2.118,10.59)_ = 3.54, *p* = 0.07 (Fig. 4*G*); CIE: RM one-way ANOVA, *F*_(2.199,19.79)_ = 1.98, *p* = 0.16 (Fig. 4*H*)]. However, when spike size was compared between groups, CIE-exposed mice showed significantly larger spikes during ethanol+quinine and ethanol+sucrose drinking (two-way ANOVA, main effects of drink: *F*_(2.382,33.35)_ = 1.39, *p* = 0.26, CIE: *F*_(1,14)_ = 0.6, *p* = 0.45, interaction: *F*_(3,42)_ = 4.8, *p* = 0.0058, Sidak’s multiple comparisons test was not significant for any drinking solution; Fig. 4*I*). These data suggest that CIE exposure alters the activity of LOFC neurons after consuming alcohol with different gustatory properties.

### LOFC activity during lever pressing

**Figure 5.**
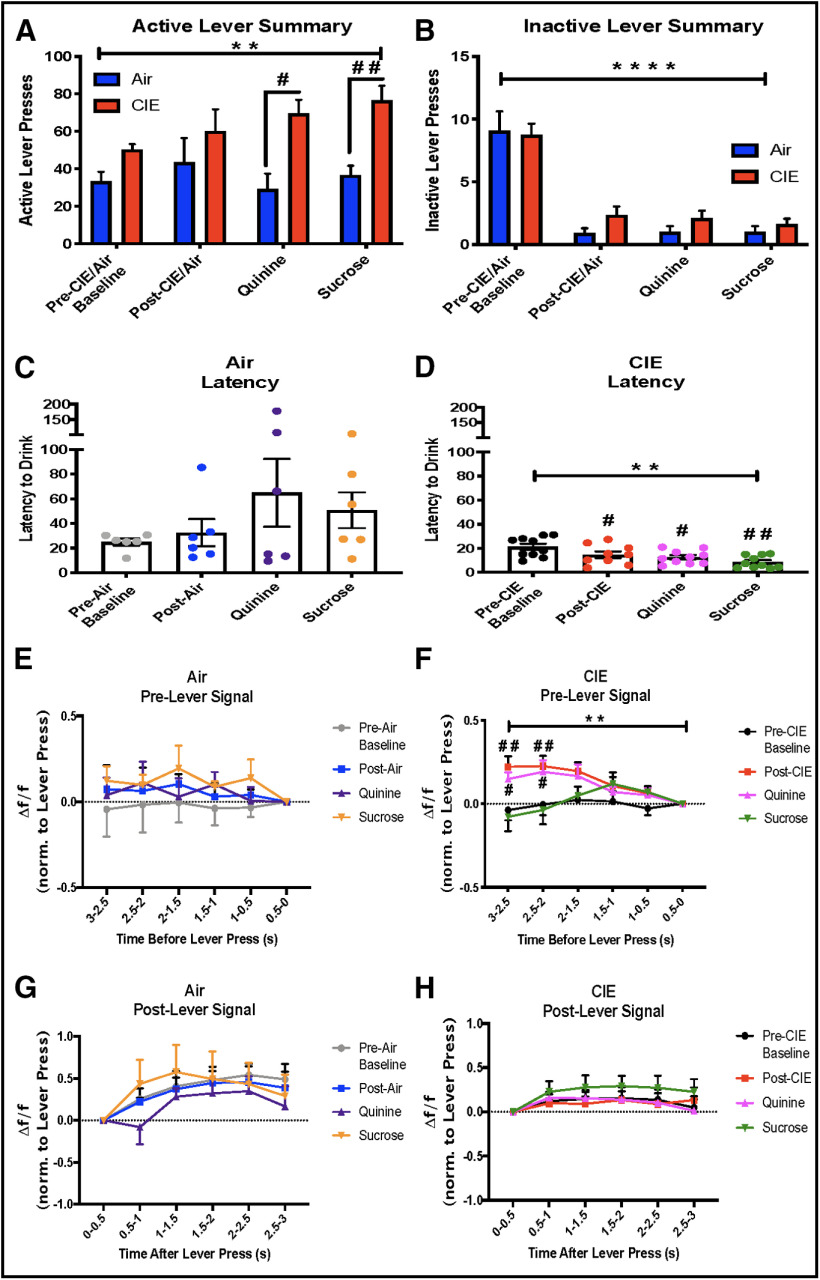
LOFC activity during lever pressing. ***A***, Compared with air-exposed mice, CIE-exposed mice had significantly more lever presses during ethanol+quinine and ethanol+sucrose drinking sessions. ***B***, Inactive lever pressing was not significantly different between air-exposed and CIE-exposed mice during any of the drinking sessions, but there was a main effect of drinking session. ***C***, Latency between a lever press and the initiation of a drinking bout was not significantly different across drinking sessions in air-exposed mice. ***D***, In CIE-exposed mice, the latency between a lever press and initiation of a drinking bout was significantly shorter during ethanol, ethanol+quinine, and ethanol+sucrose drinking sessions than baseline drinking sessions. ***E***, In air-exposed mice, there were no differences in LOFC GCaMP6f activity preceding the lever press during any drinking sessions. ***F***, In CIE-exposed mice, there was an increase in LOFC GCaMP6f activity preceding a lever press following CIE exposure during ethanol or ethanol+quinine drinking. ***G***, In air-exposed mice, there was no difference in LOFC GCaMP6f activity immediately after a lever press during any drinking session. ***H***, In CIE-exposed mice, there was no difference in LOFC activity immediately after a lever press during any drinking session; * indicates significant main effects or interactions **p < 0.01, ****p < 0.0001, # used for significant *post hoc* tests #*p* < 0.05, ##*p* < 0.01.

Upon pressing the active lever, mice were presented with both auditory (1.5-s tone) and visual cues (house light off for 1.5 s) followed by delivery of 20 μl of solution in the drinking well. Mice quickly learned to press the active lever to gain access to ethanol containing solutions. Consistent with the changes in drinking noted above, CIE-exposed mice had more active lever presses compared with air-exposed mice during ethanol+quinine and ethanol+sucrose drinking (two-way ANOVA, main effect of CIE: *F*_(1,14)_ = 15.34, *p* = 0.002, drink: *F*_(1.737,24.32)_ = 1.16, *p* = 0.32, interaction: *F*_(3,42)_ = 1.38, *p* = 0.26, Sidak’s multiple comparisons test revealed significant differences during quinine: *t* = 3.64, *p* = 0.014, and sucrose drinking: *t* = 4.32, *p* = 0.0029; Fig. 5*A*). There were no differences in inactive lever responding for either group (two-way ANOVA, main effects of CIE: *F*_(1,14)_ = 1.13, *p* = 0.31, drink: *F*_(2.362,33.07)_ = 55.35, *p* < 0.0001, interaction: *F*_(3,42)_ = 0.60, *p* = 0.62; Sidak’s multiple comparisons test > 0.05; Fig. 5*B*). Interestingly, CIE-exposed mice showed a decreased latency between the initiation of a lever press and the beginning of a drinking bout (RM one-way ANOVA, *F*_(1.718,14.89)_ = 12.06, *p* = 0.0011, Dunnett’s multiple comparisons test was significant across all drinks, post-CIE: q = 3.55, *p* = 0.019, quinine: q = 3.35, *p* = 0.022, and sucrose: q = 4.43, *p* = 0.0043; Fig. 5*D*). Conversely, latencies to drink in air-exposed mice were not significantly different across any drinking solution (RM one-way ANOVA, *F*_(1.327,6.637)_ = 1.94, *p* = 0.21; Fig. 5*C*). Examination of GCaMP6f signals of LOFC neurons of air-exposed mice surrounding the lever press revealed no significant differences in LOFC activity before (RM two-way ANOVA, main effects of time: *F*_(2.499,49.98)_ = 0.72, *p* = 0.52, drink: *F*_(3,20)_ = 0.51, *p* = 0.68, interaction: *F*_(15,100)_ = 0.41, *p* = 0.97; Fig. 5*E*) or after the lever press (RM two-way ANOVA, main effects of time: *F*_(2.918,58.37)_ = 8.47, *p* < 0.001, drink: *F*_(3,20)_ = 0.36, *p* = 0.77, interaction: *F*_(15,100)_ = 0.59 *p* = 0.87; Dunnett’s multiple comparisons test > 0.05; Fig. 5*G*). However, in CIE-treated mice, there was a significant difference in LOFC activity that preceded the lever press with an increase occurring 2–3 s before pressing the lever during sessions of ethanol or ethanol+quinine drinking (RM two-way ANOVA, main effects of time: *F*_(2.291,82.46)_ = 3.51, *p* = 0.029, drink: *F*_(3,36)_ = 2.94, *p* = 0.046, interaction: *F*_(15,180)_ = 2.62, *p* = 0.001, Dunnett’s multiple comparisons test revealed significant differences during post-CIE drinking at the earliest two time points 3–2.5 s: q = 2.95, *p* = 0.0028 and 2.5–2 s: q = 2.63, *p* = 0.0094, and quinine drinking at the earliest two time points 3–2.5 s: q = 2.40, *p* = 0.046 and 2.5–2 s: q = 2.52, *p* = 0.033; Fig. 5*F*). There were no significant differences in LOFC activity following the lever press in CIE-treated mice (RM two-way ANOVA, main effects of time: *F*_(2.17,78.11)_ = 3.13, *p* = 0.045, drink: *F*_(3,36)_ = 0.54, *p* = 0.66, interaction: *F*_(15,180)_ = 0.33, *p* = 0.99; Dunnett’s multiple comparisons test > 0.05; Fig. 5*H*). Together, these data suggest that CIE treatment may sharpen an anticipatory signal from LOFC neurons that predicts a subsequent lever press.

### CIE increases LOFC activity

**Figure 6.**
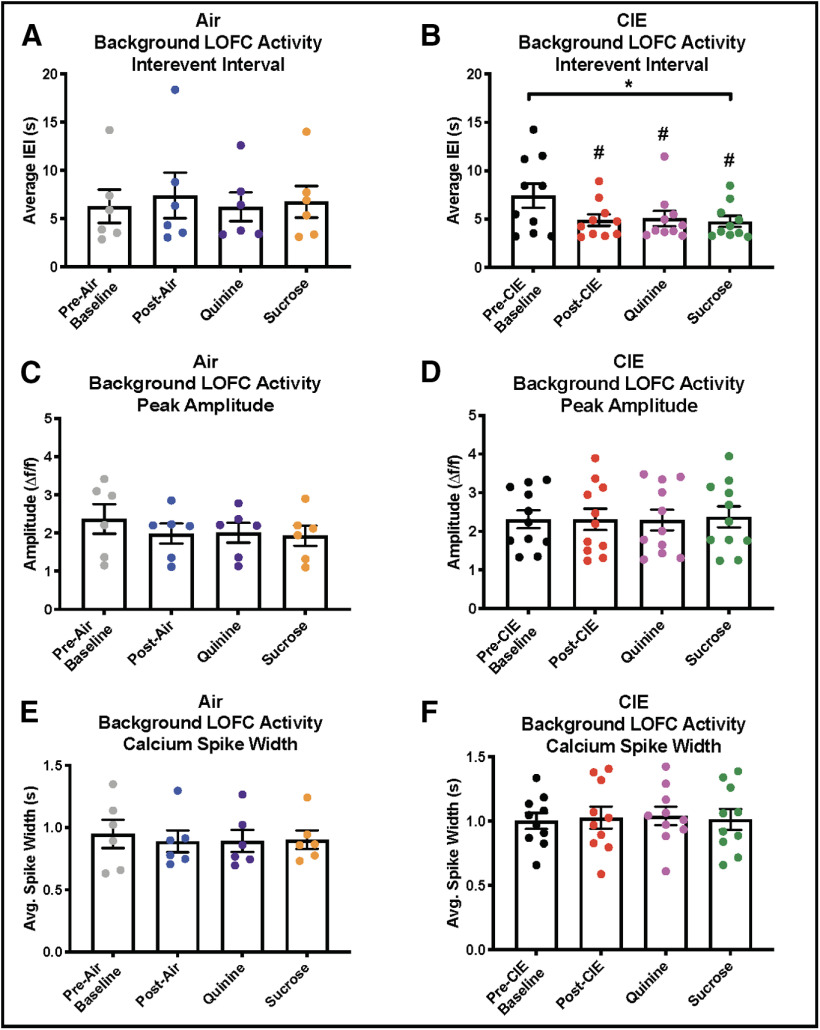
Background LOFC activity during drinking sessions. ***A***, In air-exposed mice, there was no difference in the interevent interval of GCaMP6f calcium transients during any of the drinking sessions. ***B***, In CIE-exposed mice, interevent intervals of GCaMP6f calcium transients were significantly shorter following CIE exposure during all drinking sessions relative to pre-CIE activity. ***C***, In air-exposed mice, there were no differences in the amplitude of GCaMP6f calcium transients during any drinking session. ***D***, In CIE-exposed mice, there were no differences in the amplitude of GCaMP6f calcium transients during any drinking session. ***E***, In air-exposed mice, there were no differences in the widths of GCaMP6f calcium transients during any drinking session. ***F***, In CIE-exposed mice, there were no differences in the widths of GCaMP6f calcium transients during any drinking session; * indicates significant main effect *p < 0.05, # used for significant *post hoc* test, #*p* < 0.05.

We have previously shown using slice electrophysiology that CIE exposure enhances the intrinsic excitability of LOFC neurons (Nimitvilai et al., 2016, 2018). To determine whether a similar phenomenon is observed *in vivo*, the mean frequency of peaks in the GCaMP6f signal was measured over the entirety of each drinking session. Consistent with the *ex vivo* slice studies, CIE-exposed mice showed a higher frequency of GCaMP6f calcium spikes, lower interevent interval, relative to pre-CIE baseline values (RM one-way ANOVA, *F*_(1.442,12.97)_ = 6.98, *p* = 0.014, Dunnett’s multiple comparisons test was significant for post-CIE: q = 2.84, *p* = 0.048, quinine: q = 2.91, *p* = 0.043, and sucrose: q = 2.84, *p* = 0.048; Fig. 6*B*). In contrast, there was no difference in GCaMP6f peak frequency relative to baseline in Air-treated mice (RM one-way ANOVA, *F*_(1.772,8.858)_ = 1.20, *p* = 0.34; Fig. 6*A*). In addition, relative to the pretreatment baseline, there was no difference in the amplitude or event width of GCaMP events in Air [amplitude, RM one-way ANOVA *F*_(1.074,5.369)_ = 3.13, *p* = 0.13 (Fig. 6*C*); event width, RM one-way ANOVA, *F*_(1.413,7.063)_ = 0.50, *p* = 0.57 (Fig. 6*E*)] or CIE [amplitude, RM one-way ANOVA, *F*_(2.161,21.61)_ = 0.50, *p* = 0.63 (Fig. 6*D*); event width, RM one-way ANOVA, *F*_(1.821,16.39)_ = 0.62, *p* = 0.54 (Fig. 6*F*)] exposed animals.

## Discussion

In this study, we examined the signaling characteristics of LOFC neurons in awake behaving mice using GCaMP6f fiber photometry. We report that LOFC neurons are acutely inhibited by non-contingent alcohol administration and become hyperactive following CIE exposure. We also found that LOFC neurons are responsive during both the seeking and consummatory phases of alcohol self-administration. The findings suggest that exposure to CIE may prime the LOFC to engage in the evaluation of gustatory stimuli associated with alcohol while air-exposed animals may rely on regions outside of the LOFC to guide their changes in consummatory behavior.

There is some disagreement in the literature about the effects of acute and chronic alcohol on orbitofrontal cortex activity. Some studies measuring immediate early genes or c-Fos expression have concluded that acute ethanol exposure increases OFC activity (Knapp et al., 2001; Liu and Crews, 2015), while functional studies show that OFC neurons are inhibited by acute ethanol exposure (Badanich et al., 2013). Some of these differences may be attributable to technical differences between studies, such as route of administration (e.g., bath application, intragastric infusion, intraperitoneal injection), time after last ethanol exposure (hours vs days), specificity of effect (individual glutamatergic neurons vs whole-brain region) and preparation (*in vivo* vs *ex vivo*). Previous studies from our lab, using electrophysiological recordings from *ex vivo* slice preparations, have shown that LOFC neurons are acutely inhibited by bath application of ethanol at concentrations associated with voluntary consumption (Badanich et al., 2013; Nimitvilai et al., 2020). In order to determine whether these effects persist in glutamatergic LOFC neurons *in vivo*, we examined whether intraperitoneal injections of ethanol would inhibit LOFC activity. Using fiber photometry, we found that both locomotor stimulating (2 g/kg) and sedating (3.5 g/kg) doses of ethanol significantly reduced the frequency of GCaMP6f peaks with the higher dose inhibiting ∼80% of activity. In further agreement with previous *in vitro* studies (Nimitvilai et al., 2016; Radke et al., 2017), we found that CIE exposure and withdrawal increased LOFC activity *in vivo* as reflected by the increase in the frequency of detected GCaMP6f events.

In this study, mice showed an escalation in drinking following repeated exposures to ethanol vapor but not air. Additionally, we found that while air-exposed mice were sensitive to the devaluing effects of quinine adulteration, CIE-exposed mice were not and actually showed increased drinking of this solution relative to ethanol alone. These data are consistent with previous reports (Becker and Lopez, 2004; den Hartog et al., 2016; Rose et al., 2016). In the current study, we used a relatively low concentration of quinine (60 μm) compared with other studies (Hopf et al., 2010; Timme et al., 2020), as we have previously shown (den Hartog et al., 2016) that it clearly separates the groups without completely eliminating drinking in air-exposed mice. Interestingly, CIE-exposed but not air-exposed mice also increased consumption when sucrose was added to the alcohol solution, potentially because of the order that the solutions were presented, with sucrose following quinine. Air-exposed mice may have developed an aversion during the quinine drinking that blunted any effect of sucrose. Although additional sessions might have revealed an effect of sucrose on drinking in the air-exposed mice, CIE induced increases in drinking begin to return to control levels within 7 d following the last CIE exposure thus limiting the period of comparison between groups.

We also examined the microstructure of drinking episodes and found that CIE exposure increased the number of drinking bouts per session, consistent with some previous findings (Robinson and McCool, 2015). Interestingly, a human laboratory drinking study found that an increase in the number of drinking bouts was highly correlated with reported levels of liking a drink (Gero et al., 2019). However, Barkley-Levenson and Crabbe (2015) found that two different strains of mice, selected for high alcohol consumption, consumed high levels of alcohol through either larger bouts or a higher frequency of bouts. Indeed, others have shown that CIE treatment of C57BL/6J mice increases the size of their drinking bouts without any effect on the number of bouts (Renteria et al., 2020). However, these differences may be because of the amount of access that the mice were given; 30 min in our study and 16 h in the study by Renteria et al. (2020) or the concentration of the ethanol solution 10% in our study compared with 15% (Renteria et al., 2020). Further, the addition of sucrose to the ethanol solution in the present study increased the size of drinking bouts in both Air-exposed and CIE-exposed mice, consistent with an increased preference for the sweetened solution (Barkley-Levenson and Crabbe, 2012).

In this study, we identified a characteristic response of LOFC neurons during alcohol seeking and consumption. We demonstrated that activity increases preceding the initiation of a drinking bout, decreases during active alcohol consumption and spikes immediately after drinking. These patterns of activity in the LOFC are similar to those shown by other investigators, using *in vivo* electrophysiology, during sucrose drinking (Tremblay and Schultz, 1999; Moorman and Aston-Jones, 2014; Hernandez and Moorman, 2020). Interestingly, in the present study, exposure to CIE primed LOFC neurons to alter the magnitude of their activation in response to changes in the gustatory properties of the alcohol solution. Conversely, mice exposed to air did not have significant changes in neuronal activity surrounding alcohol drinking. Considering the important role of the LOFC in processing gustatory stimuli (Rolls, 2015), we were surprised to see that LOFC signaling was unchanged in air-exposed animals during the introduction of appetitive or aversive tastants. One possible explanation is that CIE exposure increases the salience of alcohol and its associated gustatory properties, and that the LOFC is more heavily involved in situations with higher salience. CIE-exposed mice consumed significantly more alcohol following exposure while Air-exposed mice showed a trend toward decreased drinking. Considering that the OFC appears to be especially involved in situations integrating novelty and reward (Elliott et al., 2000), LOFC neurons may be more engaged when the reward is perceived as being higher, such as when alcohol is available following the development of dependence.

A growing number of studies have reported that high concentrations of alcohol (achieved through binge models or vapor exposure) alter the structural, molecular and physiological framework of the LOFC (Coleman et al., 2011, 2014; McGuier et al., 2015) as well as behaviors that are dependent on LOFC function (Obernier et al., 2002; Badanich et al., 2011; Kroener et al., 2012; Fernandez et al., 2017). While the LOFC is acutely sensitive to ethanol even at low concentrations (Knapp et al., 2001; Badanich et al., 2013), disruption of LOFC-dependent behaviors appears to require higher levels of drinking or chronic ethanol exposure (McMurray et al., 2014, 2016; den Hartog et al., 2016). Furthermore, lesions or chemogenetic inhibition of the LOFC had no effect on drinking in air-exposed non-dependent mice but increased consumption in CIE-treated animals (den Hartog et al., 2016). These findings support the theory that high levels of alcohol exposure are necessary to disrupt LOFC function, or fully engage it in the evaluation of alcohol-related cues.

The LOFC is thought to maintain a pliable representation of the anticipated value of a reward (Yan et al., 2016). In this study, we found that CIE treatment resulted in enhanced LOFC activity in the seconds preceding active lever pressing during ethanol and ethanol+quinine drinking sessions, an effect that was not seen during baseline drinking or in air-exposed mice. Accordingly, CIE-exposed mice may have increased anticipation or motivation of obtaining the ethanol reward leading to elevated LOFC activity just before the lever press. Following CIE exposure, mice had a decreased latency between lever pressing and alcohol consumption likely reflecting a difference in reward anticipation. Interestingly, this anticipatory prelever activity returned close to baseline by the last day of testing with the ethanol+sucrose solution, although there remained a small but non-significant increase in activity during the second before lever pressing during these sessions. There are several studies that have shown that four cycles of CIE produce a number of effects which return to baseline within a week or two of the final exposure. For example, AMPA/NMDA ratios in LOFC neurons return to baseline levels by 7 d after CIE (Nimitvilai et al., 2016) and c-Fos activity in the LOFC is similar to air controls by 7 d of withdrawal (Smith et al., 2019). Also, CIE-induced escalations in drinking are attenuated the second week after the final CIE exposure (Lopez and Becker, 2005). Therefore, it is possible that the reduction in prelever activity during ethanol+sucrose drinking is related to the timing of these sessions 7 d after the last vapor exposure.

In conclusion, the results of this study show that repeated cycles of CIE exposure increase alcohol consumption, decrease the devaluing effect of quinine and produce time-locked alterations in the activity of LOFC neurons during operant ethanol self-administration. While these studies did not distinguish between projection specific subpopulations of LOFC neurons, future studies should examine how CIE alters the signaling characteristics of LOFC neurons in neural circuits involved in anticipation of reward delivery, evaluation of reward value, and implementation of flexible behavior.

## Synthesis

Reviewing Editor: Leandro Vendruscolo, The Scripps Research Institute Decisions are customarily a result of the Reviewing Editor and the peer reviewers coming together and discussing their recommendations until a consensus is reached. When revisions are invited, a fact-based synthesis statement explaining their decision and outlining what is needed to prepare a revision will be listed below. Note: If this manuscript was transferred from JNeurosci and a decision was made to accept the manuscript without peer review, a brief statement to this effect will instead be what is listed below.

There are a few minor issues that need to be addressed:

Page 4: what was the light/dark schedule in the holding rooms? Was behavior testing conducted in dark or light conditions?

Minimize the use of abbreviations. For those abbreviations that will be maintained, spell out at the first time they appear in the manuscript.

Was ethanol 10% v/v or w/v?

Post hoc comparisons should not be followed if the ANOVA was not significant. For example, page 11, second paragraph. Please correct that and adjust the discussion and figure legends as necessary.

Page 17, top paragraph: the acute effects of alcohol were presented as figure 2, whereas the discussion is presented last. Reorder the figures or the discussion.

ANOVA values are presented in the legend of Figure 2, but they are not in the other figure legends. Make it consistent.

## References

[B1] Aguayo LG, Castro P, Mariqueo T, Muñoz B, Xiong W, Zhang L, Lovinger DM, Homanics GE (2014) Altered sedative effects of ethanol in mice with α1 glycine receptor subunits that are insensitive to Gβγ modulation. Neuropsychopharmacology 39:2538–2548. 10.1038/npp.2014.100 24801766 PMC4207329

[B2] Badanich KA, Becker HC, Woodward JJ (2011) Effects of chronic intermittent ethanol exposure on orbitofrontal and medial prefrontal cortex-dependent behaviors in mice. Behav Neurosci 125:879–891. 10.1037/a0025922 22122149 PMC3229192

[B3] Badanich KA, Mulholland PJ, Beckley JT, Trantham-Davidson H, Woodward JJ (2013) Ethanol reduces neuronal excitability of lateral orbitofrontal cortex neurons via a glycine receptor dependent mechanism. Neuropsychopharmacology 38:1176–1188. 10.1038/npp.2013.12 23314219 PMC3656360

[B4] Baltz ET, Yalcinbas EA, Renteria R, Gremel CM (2018) Orbital frontal cortex updates state-induced value change for decision-making. Elife 7:e35988. 10.7554/eLife.35988 29897332 PMC6039177

[B5] Barkley-Levenson AM, Crabbe JC (2012) Ethanol drinking microstructure of a high drinking in the dark selected mouse line. Alcohol Clin Exp Res 36:1330–1339. 10.1111/j.1530-0277.2012.01749.x 22524154 PMC3407303

[B6] Barkley-Levenson AM, Crabbe JC (2015) Distinct ethanol drinking microstructures in two replicate lines of mice selected for drinking to intoxication. Genes Brain Behav 14:398–410. 10.1111/gbb.12225 25981501 PMC4749147

[B7] Becker HC, Lopez MF (2004) Increased ethanol drinking after repeated chronic ethanol exposure and withdrawal experience in C57BL/6 mice. Alcohol Clin Exp Res 28:1829–1838. 10.1097/01.alc.0000149977.95306.3a 15608599

[B8] Coleman LG Jr, He J, Lee J, Styner M, Crews FT (2011) Adolescent binge drinking alters adult brain neurotransmitter gene expression, behavior, brain regional volumes, and neurochemistry in mice. Alcohol Clin Exp Res 35:671–688. 10.1111/j.1530-0277.2010.01385.x 21223304 PMC3544413

[B9] Coleman LG Jr, Liu W, Oguz I, Styner M, Crews FT (2014) Adolescent binge ethanol treatment alters adult brain regional volumes, cortical extracellular matrix protein and behavioral flexibility. Pharmacol Biochem Behav 116:142–151. 10.1016/j.pbb.2013.11.021 24275185 PMC3913047

[B10] den Hartog C, Zamudio-Bulcock P, Nimitvilai S, Gilstrap M, Eaton B, Fedarovich H, Motts A, Woodward JJ (2016) Inactivation of the lateral orbitofrontal cortex increases drinking in ethanol-dependent but not non-dependent mice. Neuropharmacology 107:451–459. 10.1016/j.neuropharm.2016.03.031 27016020 PMC4934018

[B11] Elliott R, Dolan RJ, Frith CD (2000) Dissociable functions in the medial and lateral orbitofrontal cortex: evidence from human neuroimaging studies. Cereb Cortex 10:308–317. 10.1093/cercor/10.3.308 10731225

[B12] Fernandez GM, Lew BJ, Vedder LC, Savage LM (2017) Chronic intermittent ethanol exposure leads to alterations in brain-derived neurotrophic factor within the frontal cortex and impaired behavioral flexibility in both adolescent and adult rats. Neuroscience 348:324–334. 10.1016/j.neuroscience.2017.02.045 28257889 PMC5458357

[B13] Gardner MPH, Conroy JS, Shaham MH, Styer CV, Schoenbaum G (2017) Lateral orbitofrontal inactivation dissociates devaluation-sensitive behavior and economic choice. Neuron 96:1192–1203.e4. 10.1016/j.neuron.2017.10.026 29154127 PMC5728681

[B14] Gero D, File B, Justiz J, Steinert RE, Frick L, Spector AC, Bueter M (2019) Drinking microstructure in humans: a proof of concept study of a novel drinkometer in healthy adults. Appetite 133:47–60. 10.1016/j.appet.2018.08.012 30179650

[B15] Gourley SL, Olevska A, Zimmermann KS, Ressler KJ, Dileone RJ, Taylor JR (2013) The orbitofrontal cortex regulates outcome-based decision-making via the lateral striatum. Eur J Neurosci 38:2382–2388. 10.1111/ejn.12239 23651226 PMC3864662

[B16] Hamilton DA, Brigman JL (2015) Behavioral flexibility in rats and mice: contributions of distinct frontocortical regions. Genes Brain Behav 14:4–21. 10.1111/gbb.12191 25561028 PMC4482359

[B17] Hernandez JS, Moorman DE (2020) Orbitofrontal cortex encodes preference for alcohol. eNeuro 7:ENEURO.0402-19.2020. 10.1523/ENEURO.0402-19.2020 PMC7365858 32661066

[B18] Hopf FW, Chang SJ, Sparta DR, Bowers MS, Bonci A (2010) Motivation for alcohol becomes resistant to quinine adulteration after 3 to 4 months of intermittent alcohol self-administration. Alcohol Clin Exp Res 34:1565–1573. 10.1111/j.1530-0277.2010.01241.x 20586757 PMC2997761

[B19] King CE, Griffin WC, Luderman LN, Kates MM, McGinty JF, Becker HC (2017) Oxytocin reduces ethanol self-administration in mice. Alcohol Clin Exp Res 41:955–964. 10.1111/acer.13359 28212464 PMC5404956

[B20] Knapp DJ, Braun CJ, Duncan GE, Qian Y, Fernandes A, Crews FT, Breese GR (2001) Regional specificity of ethanol and NMDA action in brain revealed with FOS-like immunohistochemistry and differential routes of drug administration. Alcohol Clin Exp Res 25:1662–1672. 11707641

[B21] Kroener S, Mulholland PJ, New NN, Gass JT, Becker HC, Chandler LJ (2012) Chronic alcohol exposure alters behavioral and synaptic plasticity of the rodent prefrontal cortex. PLoS One 7:e37541. 10.1371/journal.pone.0037541 22666364 PMC3364267

[B22] Lerner TN, Shilyansky C, Davidson TJ, Evans KE, Beier KT, Zalocusky KA, Crow AK, Malenka RC, Luo L, Tomer R, Deisseroth K (2015) Intact-brain analyses reveal distinct information carried by SNc dopamine subcircuits. Cell 162:635–647. 10.1016/j.cell.2015.07.014 26232229 PMC4790813

[B23] Liu W, Crews FT (2015) Adolescent intermittent ethanol exposure enhances ethanol activation of the nucleus accumbens while blunting the prefrontal cortex responses in adult rat. Neuroscience 293:92–108. 10.1016/j.neuroscience.2015.02.014 25727639 PMC4821202

[B24] Lopez MF, Becker HC (2005) Effect of pattern and number of chronic ethanol exposures on subsequent voluntary ethanol intake in C57BL/6J mice. Psychopharmacology (Berl) 181:688–696. 10.1007/s00213-005-0026-3 16001125

[B25] Lopez MF, Becker HC, Chandler LJ (2014) Repeated episodes of chronic intermittent ethanol promote insensitivity to devaluation of the reinforcing effect of ethanol. Alcohol 48:639–645. 10.1016/j.alcohol.2014.09.002 25266936 PMC4250386

[B501] McGuier NS, Padula AE, Lopez MF, Woodward JJ, Mulholland PJ (2015) Withdrawal from chronic intermittent alcohol exposure increases dendritic spine density in the lateral orbitofrontal cortex of mice. Alcohol 49:21–27. 25468278 10.1016/j.alcohol.2014.07.017 PMC4314373

[B26] McMurray MS, Amodeo LR, Roitman JD (2014) Effects of voluntary alcohol intake on risk preference and behavioral flexibility during rat adolescence. PLoS One 9:e100697. 10.1371/journal.pone.0100697 25007338 PMC4090063

[B27] McMurray MS, Amodeo LR, Roitman JD (2016) Consequences of adolescent ethanol consumption on risk preference and orbitofrontal cortex encoding of reward. Neuropsychopharmacology 41:1366–1375. 10.1038/npp.2015.288 26370327 PMC4793121

[B28] Moorman DE, Aston-Jones G (2014) Orbitofrontal cortical neurons encode expectation-driven initiation of reward-seeking. J Neurosci 34:10234–10246. 10.1523/JNEUROSCI.3216-13.2014 25080585 PMC4115135

[B29] Nimitvilai S, Lopez MF, Mulholland PJ, Woodward JJ (2016) Chronic intermittent ethanol exposure enhances the excitability and synaptic plasticity of lateral orbitofrontal cortex neurons and induces a tolerance to the acute inhibitory actions of ethanol. Neuropsychopharmacology 41:1112–1127. 10.1038/npp.2015.250 26286839 PMC4748436

[B30] Nimitvilai S, Lopez MF, Woodward JJ (2018) Effects of monoamines on the intrinsic excitability of lateral orbitofrontal cortex neurons in alcohol-dependent and non-dependent female mice. Neuropharmacology 137:1–12. 10.1016/j.neuropharm.2018.04.019 29689260 PMC6050070

[B31] Nimitvilai S, Lopez MF, Woodward JJ (2020) Sex-dependent differences in ethanol inhibition of mouse lateral orbitofrontal cortex neurons. Addict Biol 25:e12698. 10.1111/adb.12698 30468275 PMC8168337

[B32] Nurmi M, Kiianmaa K, Sinclair JD (1994) Brain ethanol in AA, ANA, and Wistar rats monitored with one-minute microdialysis. Alcohol 11:315–321. 10.1016/0741-8329(94)90098-1 7945986

[B33] Obernier JA, White AM, Swartzwelder HS, Crews FT (2002) Cognitive deficits and CNS damage after a 4-day binge ethanol exposure in rats. Pharmacol Biochem Behav 72:521–532. 10.1016/s0091-3057(02)00715-3 12175448

[B34] Padoa-Schioppa C, Assad JA (2006) Neurons in the orbitofrontal cortex encode economic value. Nature 441:223–226. 10.1038/nature04676 16633341 PMC2630027

[B35] Panayi MC, Killcross S (2018) Functional heterogeneity within the rodent lateral orbitofrontal cortex dissociates outcome devaluation and reversal learning deficits. Elife 7:e37357. 10.7554/eLife.37357 30044220 PMC6101941

[B36] Radke AK, Jury NJ, Kocharian A, Marcinkiewcz CA, Lowery-Gionta EG, Pleil KE, McElligott ZA, McKlveen JM, Kash TL, Holmes A (2017) Chronic EtOH effects on putative measures of compulsive behavior in mice. Addict Biol 22:423–434. 10.1111/adb.12342 26687341 PMC4916036

[B37] Renteria R, Cazares C, Gremel CM (2020) Habitual ethanol seeking and licking microstructure of enhanced ethanol self-administration in ethanol-dependent mice. Alcohol Clin Exp Res 44:880–891. 10.1111/acer.14302 32020644 PMC7238766

[B38] Riceberg JS, Shapiro ML (2017) Orbitofrontal cortex signals expected outcomes with predictive codes when stable contingencies promote the integration of reward history. J Neurosci 37:2010–2021. 10.1523/JNEUROSCI.2951-16.2016 28115481 PMC5338752

[B39] Robinson SL, McCool BA (2015) Microstructural analysis of rat ethanol and water drinking patterns using a modified operant self-administration model. Physiol Behav 149:119–130. 10.1016/j.physbeh.2015.05.034 26037631 PMC4506870

[B40] Rolls ET (2015) Taste, olfactory, and food reward value processing in the brain. Prog Neurobiol 127-128:64–90. 10.1016/j.pneurobio.2015.03.002 25812933

[B41] Rose JH, Karkhanis AN, Chen R, Gioia D, Lopez MF, Becker HC, McCool BA, Jones SR (2016) Supersensitive kappa opioid receptors promotes ethanol withdrawal-related behaviors and reduce dopamine signaling in the nucleus accumbens. Int J Neuropsychopharmacol 19:pyv127. 26625893 10.1093/ijnp/pyv127 PMC4886667

[B42] Schoenbaum G, Chiba AA, Gallagher M (1998) Orbitofrontal cortex and basolateral amygdala encode expected outcomes during learning. Nat Neurosci 1:155–159. 10.1038/407 10195132

[B43] Schoenbaum G, Nugent SL, Saddoris MP, Setlow B (2002) Orbitofrontal lesions in rats impair reversal but not acquisition of go, no-go odor discriminations. Neuroreport 13:885–890. 11997707 10.1097/00001756-200205070-00030

[B44] Schoenbaum G, Setlow B, Saddoris MP, Gallagher M (2003) Encoding predicted outcome and acquired value in orbitofrontal cortex during cue sampling depends upon input from basolateral amygdala. Neuron 39:855–867. 10.1016/s0896-6273(03)00474-4 12948451

[B45] Smith RJ, Anderson RI, Haun HL, Mulholland PJ, Griffin WC 3rd, Lopez MF, Becker HC (2019) Dynamic c-Fos changes in mouse brain during acute and protracted withdrawal from chronic intermittent ethanol exposure and relapse drinking. Addict Biol 25:e12804. 31288295 10.1111/adb.12804 PMC7579841

[B46] Smoothy R, Berry MS (1985) Time course of the locomotor stimulant and depressant effects of a single low dose of ethanol in mice. Psychopharmacology (Berl) 85:57–61. 10.1007/BF00427322 3920699

[B47] Teitelbaum H (1964) A comparison of effects of orbitofrontal and hippocampal lesions upon discrimination learning and reversal in the cat. Exp Neurol 9:452–462. 10.1016/0014-4886(64)90053-6 14188532

[B48] Timme NM, Linsenbardt D, Timm M, Galbari T, Cornwell E, Lapish C (2020) Alcohol-preferring P rats exhibit aversion-resistant drinking of alcohol adulterated with quinine. Alcohol 83:47–56. 10.1016/j.alcohol.2019.09.003 31542609 PMC7064416

[B49] Tremblay L, Schultz W (1999) Relative reward preference in primate orbitofrontal cortex. Nature 398:704–708. 10.1038/19525 10227292

[B50] West EA, DesJardin JT, Gale K, Malkova L (2011) Transient inactivation of orbitofrontal cortex blocks reinforcer devaluation in macaques. J Neurosci 31:15128–15135. 10.1523/JNEUROSCI.3295-11.2011 22016546 PMC3224797

[B51] Yan C, Su L, Wang Y, Xu T, Yin DZ, Fan MX, Deng CP, Hu Y, Wang ZX, Cheung EF, Lim KO, Chan RC (2016) Multivariate neural representations of value during reward anticipation and consummation in the human orbitofrontal cortex. Sci Rep 6:29079. 10.1038/srep29079 27378417 PMC4932626

[B52] Zamudio PA, Gioia DA, Lopez M, Homanics GE, Woodward JJ (2021) The escalation in ethanol consumption following chronic intermittent ethanol exposure is blunted in mice expressing ethanol-resistant GluN1 or GluN2A NMDA receptor subunits. Psychopharmacology (Berl) 238:271–279. 10.1007/s00213-020-05680-z 33052417 PMC7796987

